# Integrated Proteomic and Molecular Identification of Thermophilic *Geobacillus* Strains from Algerian Desert Sands and Their Enzymatic Potential

**DOI:** 10.3390/life15081327

**Published:** 2025-08-21

**Authors:** Amaria Ilhem Hammadi, Mohamed Merzoug, Marwa Aireche, Zohra Yasmine Zater, Keltoum Bendida, Chaimaa Naila Brakna, Slimane Choubane, Svetoslav Dimitrov Todorov, Djamal Saidi

**Affiliations:** 1 Higher School of Biological Sciences of Oran, BP 1042 Saim Mohamed, Cité Emir Abdelkader (EX-INESSMO), Oran 31000, Algeria; hammadiamaria267@gmail.com (A.I.H.); marwa.ar231@gmail.com (M.A.); keltoumbendida2001@gmail.com (K.B.); chaimaa1012@gmail.com (C.N.B.); choubane.slimane@gmail.com (S.C.); djamsaidi@gmail.com (D.S.); 2 Laboratory of Biology of Microorganisms and Biotechnology, University of Oran1 Ahmed Ben Bella, Oran 31005, Algeria; yassminezater93@gmail.com; 3 ProBacLab, Laboratório de Microbiologia de Alimentos, Departamento de Alimentos e Nutrição Experimental, Food Research Center, Faculdade de Ciências Farmacêuticas, Universidade de São Paulo, São Paulo 05508-000, SP, Brazil; 4 Department of General Hygiene, I.M. Sechenov First Moscow State Medical University, Trubetskaya St., Bldg. 8/2, Moscow 119435, Russia

**Keywords:** sandy soil, *Geobacillus*, thermophiles, thermostable enzymes, MALDI-TOF MS, 16S rRNA

## Abstract

Thermophilic microorganisms are among the key natural sources of thermostable enzymes, found not only in geothermal areas but also in arid environments. In this study, eight *Geobacillus* strains were isolated from the arid sands of Aïn Sefra (Naâma, Algeria) and characterized both phenotypically and genetically. All strains exhibited an optimal growth temperature of 70 °C, with most showing alkaliphilic pH preferences. Proteomic and molecular analyses (MALDI-TOF MS, 16S rRNA) identified *Geobacillus kaustophilus* as predominant, with BOX-PCR and RAPD-PCR revealing notable intraspecies diversity. All strains synthesized at least one thermostable enzyme (protease, amylase, laccase, or DNase) at their optimal temperature (70 °C), positioning them as promising candidates for biotechnological processes requiring extreme thermal conditions.

## 1. Introduction

Thermophiles, a class of extremophiles, have garnered significant research interest due to their exceptional thermos-resistance and unique macromolecular adaptations. These properties enable them to thrive at high temperatures, exhibiting robust metabolic activity, chemically and physically stable enzymes, and enhanced productivity despite slower growth rates compared to mesophilic counterparts [1,2]. Thermophiles are predominantly isolated from a variety of extreme environments, including hot springs, geothermal areas as well as from mesobiotic habitats such as soils, compost piles. They are classified into three groups based on their optimal growth temperatures: moderate thermophiles (50–60 °C), extreme thermophiles (60–80 °C) and hyperthermophiles (80–110 °C) [3,4], with extreme thermophiles being the most extensively studied due to their relative ease of manipulation, particularly those encompassing aerobic strains. Their requirement for elevated growth temperatures makes them of significant biotechnological interest, as they harbor a repertoire of thermostable biomolecules, particularly thermozymes, which exhibit remarkable stability under high-temperature conditions [5].

The genus *Geobacillus* comprises predominantly aerobic or facultatively anaerobic bacteria capable of growth at temperatures between 45 and 80 °C [6]. Their thermotolerance renders them valuable reservoirs for the production of thermostable enzymes, including proteases, amylases, DNA polymerases, and others, which exhibit unique properties suited for high-temperature biotechnological processes [7], for example, proteases [8,9] are widely employed in detergents [10] and food processing [11]. Similarly, laccases [12] find applications in biomedicine and bioremediation [13]. Additionally, *Geobacillus* derived amylases [14] are utilized in starch processing, paper manufacturing, and biodegradation [13,15]. Their industrial relevance is attributed to key properties such as enhanced thermal stability, allowing operation under high-temperature conditions, accelerated reaction rates at elevated temperatures, which improve process efficiency, and reduced risks of microbial contamination, as thermophilic environments inhibit the growth of mesophilic pathogens [16]. Furthermore, they are stable in the presence of detergents, organic solvents, and in a broad range of pH conditions, including both acidic and alkaline environments [4].

In recent decades, the taxonomy and characterization of thermophilic bacteria have garnered significant attention due to their unique physiological adaptations and potential biotechnological applications. However, a substantial portion of bacterial diversity in natural environments remains unexplored, representing a vast reservoir of untapped biotechnological potential [7,17] Recent research has provided compelling evidence for the reclassification of thermophilic *Bacillus* species into multiple new genera, including *Amphibacillus*, *Alicyclobacillus*, *Paenibacillus*, *Aneurinibacillus*, *Brevibacillus*, *Halobacillus*, *Virgibacillus*, *Gracilibacillus*, *Sulfobacillus*, *Salibacillus*, *Anoxybacillus*, *Coprobacillus*, *Thermobacillus*, *Filobacillus*, *Geobacillus*, *Ureibacillus*, *Jeotgalibacillus*, and *Marinibacillus* [18]. This advancement, driven by 16S rRNA sequence analysis, has significantly enhanced the ability to identify and characterize thermophilic microorganisms at both species and subspecies levels. This method has also been used to study ecosystem diversity, reveal phylogenetic relationships between strains, and distinguish closely related microorganisms. Alongside 16S rRNA sequencing, additional molecular techniques, such as repetitive-element PCR (rep-PCR) and randomly amplified polymorphic DNA (RAPD-PCR), have further contributed to analyzing ecosystem diversity, elucidate phylogenetic relationships between strains, and differentiate microorganisms that are genetically closely related [19,20].

This study aims to isolate and characterize aerobic, spore-forming thermophilic *Geobacillus* strains from the unexplored sand ecosystem of Aïn Sefra (Naâma, Algeria) for the first time, using a proteomic and a molecular approach for strain identification, along with the screening of their capacity to produce thermostable enzymes, with high biotechnological potential particularly in processes requiring high-temperature stability.

## 2. Materials and Methods

Selection of the collection site:

Sampling was conducted in September 2023 in Aïn Sefra (Naâma Province), at the southern edge of the High Plateaus, in the Ksour Mountains of the Saharan Atlas (western Algeria). The region features a gradient in rainfall from less than 150 mm to over 350 mm annually and comprises steppe, mountainous, and pre-Saharan zones. The sampling took place in the pre-Saharan zone, characterized by harsh conditions with sparse vegetation and a semi-arid climate [21].

The map in Supplementary Figure S1 from desert ecosystem of Aïn Sefra, Naâma Province, Algeria displays the locations of the sampling sites in this study.

Seven individual sand dune samples were collected aseptically from 10 cm depth at the study site (32°44′18.0″ N 0°34′59.0″ W) using sterile spatulas, pooled in a sterile container, and homogenized for thermophiles isolation. The in-situ temperature at the collection site ranged between 53.5 °C and 61.4 °C, as measured using a Mini Thermometer IR Extech IR400 pocket device (Extech Instruments, Nashua, NH, USA). Samples were maintained at 4 °C during transportation and subsequently stored at the same temperature in the microbiology laboratory of the Genomics Technology Platform of the Higher School of Biological Sciences of Oran (PLAGENOR) until further analysis.

To isolate thermophilic bacteria, a modified liquid medium (MLM) was formulated inspired by standard enrichment culture techniques. The medium contained 0.4% (*w*/*v*) mannitol (Biochem, Cosne-Cours-sur-Loire, France), 0.4% (*w*/*v*) nutrient broth (Conda, Madrid, Spain), 0.025% (*w*/*v*) K_2_HPO_4_ (SPECILAB, Algiers, Algeria), 0.025% (*w*/*v*) yeast extract (Liofilchem, Roseto degli Abruzzi, Italy), 0.005% (*w*/*v*) NaCl (SPECILAB, Algiers, Algeria), and 0.05% (*w*/*v*) MgSO_4_ (VWR Chemicals, Radnor, PA, USA). Ten grams of sand was inoculated into 100 mL of MLM and incubated at 70 °C for 48 h under shaking at 150 rpm to obtain a homogenized soil suspension.

Subsequently, 100 µL of the culture was streaked onto modified solid medium (MSM), as described for MLM, supplemented with 2% agar (Liofilchem, Roseto degli Abruzzi, Italy). The plates were incubated at 70 °C for 24 h to isolate thermophilic aerobic bacteria.

The first eight pure strains obtained through successive subculturing on MSM plates were cryopreserved in (*v*/*v*) glycerol at −20 °C and subsequently utilized for all experimental analyses in this study.

Characterization of selected isolates:

The characterization of each isolate was conducted through the analysis of colony morphology, Gram staining, and the determination of endospore formation. Additionally, physiological properties such as optimal temperature and pH for growth were evaluated according to [22].

Morphological properties:

The morphological characteristics of purified bacterial isolates were assessed using 24 h cultures grown on MSM at 70 °C. Colony parameters such as form, texture, color, elevation, margin, surface opacity, and pigmentation were recorded [23]. Gram staining was performed following standard protocols using a light microscope B-510DK—Optika Microscopes (Optika, Ponteranica, Italy) to determine cell morphology and Gram staining [24].

Sporulation test:

To confirm endospore formation, saturated cultures were subjected to heat treatment at 100 °C for 30 min to eliminate vegetative cells. Subsequently, 100 μL of the boiled culture was inoculated into fresh MLM and incubated at 70 °C for 48 h. Growth was monitored by measuring optical density (OD) at 600 nm using a UV-vis spectrophotometer (UV-1900i—Shimadzu Europa Analytical Instruments, Duisburg, Germany) [1].

Effect of temperature and pH on strain growth:

The physiological characterization of thermophilic isolates was performed following modified protocols based on Rafiee et al. [25]. All strains were cultured aerobically in MLM for 48 h to determine their optimal growth temperature (tested range: 37 to 80 °C at culture media pH 8.4) and pH tolerance (tested range: 3.0 to11.0 at 70 °C). Growth was monitored by measuring optical density at 600 nm (OD_600_). All experimental conditions were assessed in triplicate to ensure reproducibility.

Matrix-assisted laser desorption ionization–time of flight mass spectrometry (MALDI-TOF MS) for bacterial identification:

Bacterial strains were pre-identified using the MALDI Biotyper^®^ Sirius (Bruker Daltonics GmbH & Co. KG, Bremen, Germany) System by direct colony method following the established protocol. Each pure colony was cultivated on MSM at 70 °C for 24 h. After incubation, the colonies were spread in a thin layer onto an MSP 96 target polished steel BC plate (Bruker Daltonik, Bremen, Germany). Cell lysis was induced by adding 1 μL of 70% formic acid (Prolabo, VWR International, Fontenay-sous-Bois, France), followed by air drying at room temperature. Bacterial components were crystallized by applying 1 μL of a saturated α-cyano-4-hydroxycinnamic acid (HCCA) matrix (Bruker Daltonics GmbH & Co. KG, Bremen, Germany), which was dissolved in Bruker Daltonik standard solvent, and allowed to dry at room temperature. The MSP target was inserted into the instrument according to the manufacturer’s instructions. The spectral profiles of each isolate were compared to those in the Bruker Biotyper database, and identification was assigned based on a reliability score. Prior to the analysis, the MALDI-TOF calibration standard (BTS: bacterial test standard), (Bruker Daltonics GmbH & Co. KG, Bremen, Germany) was tested to ensure proper instrument calibration and validate the accuracy of the obtained results.

According to the established criteria, a score ≥ 2.0 signified a highly probable species identification, while a score ranging from 1.7 to 2.0 indicated identification at the genus level. Scores below 1.7 were considered unreliable, classifying the isolate as unidentified [26].

Genomic profiling:

To provide additional support to our proteomic analysis, genotyping of the isolates was performed using two distinct methods to differentiate and characterize the genetic variation among the strains based on their genetic content. This happened after DNA extraction using DNA extraction kit (Invitrogen Life Technologies, Carlsbad, CA, USA) and quantification through ScanDrop2 (Analytik Jena, Jena, Germany).

BOX PCR:

The genetic similarity among the eight bacterial strains was assessed using the BOX-PCR fingerprinting technique with the BOX A1R primer (5′-CTA CGG CAA GGC GAC GCT GAC G-3′). The primer was synthesized at the PLAGENOR genomic platform and used to amplify BOX-like elements within the bacterial genomes [27,28]. The PCR reaction (30 µL) was prepared using 2× PCR Master Mix (Thermo Fisher Scientific, Waltham, MA, USA), 3 µL of BOX A1R primer (10 µM), 1.5 µL of BSA (20 mg/mL), 1 µL of genomic DNA (~60 ng), and nuclease-free water. Amplification was performed following the protocol described in Adiguzel et al. [27].

Random Amplified Polymorphic DNA PCR (RAPD) analysis:

The RAPD-PCR reaction was carried out in a 25 µL reaction mixture containing 1–5 µL of DNA (~60 ng), 12.5 µL of DreamTaq PCR Master Mix (2×) (Thermo Scientific™, Thermo Fisher Scientific, Waltham, MA, USA), and 1 µL of the OPR13 primer (5′-GGA CGA CAA G-3′) [28] synthesized at PLAGENOR genomic platform. Amplification conditions were prepared as follows: an initial denaturation step at 94 °C for 3 min and 45 s followed by 35 cycles of denaturation at 94 °C for 15 s, annealing at 36 °C for 15 s, extension at 72 °C for 2 min, with a final extension step at 72 °C for 4 min [29].

The resulting PCR products (20 µL) from both PCR experimental approaches were separated by gel electrophoresis on a 1.5% (*w*/*v*) agarose gel in 1× TAE buffer for 90 min, stained with SYBR™ Safe DNA Gel Stain (Invitrogen™, Thermo Fisher Scientific, Waltham, MA, USA), and visualized using the ChemiDoc MP System (Bio-Rad laboratories, Hercules, CA, USA).

Taxonomic identification of the isolates:

The taxonomic identification of the isolates was performed through the amplification of the 16S rRNA gene, a widely used molecular marker for bacterial classification.

The 16S rRNA gene was amplified from genomic DNA using universal primers (forward primer: 27F = 5′-AGA GTT TGA TCC TGG CTC AG-3′; reverse primer: 1492R = 5′-GGT TAC CTT GTT ACG ACT T-3′) [30]. The amplification reaction was carried out in a final volume of 50 µL, containing 25 µL of Thermo Scientific™ DreamTaq PCR Master Mix (2×), 1 µL of each primer, 1–5 µL of genomic DNA, and nuclease-free water to adjust the volume. The PCR was performed in SimpliAmp™ Thermal Cycler (Applied Biosystems™, Thermo Fisher Scientific, Waltham, MA, USA) under the following conditions: initial denaturation at 95 °C for 3 min, followed by 30 cycles of denaturation at 95 °C for 30 s, annealing at 54 °C for 30 s, extension at 72 °C for 1 min, and a final polymerization step of 72 °C for 5 min. The PCR products were analyzed on a 1% agarose gel stained with Invitrogen™ SYBR™ Safe DNA Gel Stain, alongside a GeneRuler Express DNA Ladder (Thermo Scientific™, Thermo Fisher Scientific, Waltham, MA, USA) for size comparison. The amplified products were purified using ExoSAP-IT™ Express PCR Product Cleanup Kit (Applied Biosystems™, Thermo Fisher Scientific, Waltham, MA, USA) and sent for sequencing using Big Dye Chemistry with some modifications at the PLAGENOR genomics platform, using the 3500 Genetic Analyzer (Applied Biosystems, Foster City, CA, USA).

The resulting sequences were analyzed, scrutinized and adjusted using Chromas (Technelysium DNA Sequencing Software 2.6.6) and SnapGene viewer version 8. The corrected nucleotide sequences were compared to known sequences in the NCBI database using the BLAST algorithm (version 2.15.0) (https://www.ncbi.nlm.nih.gov (accessed on 1 March 2025)) [31] and EzBioCloud (https://www.ezbiocloud.net) (accessed on 1 March 2025)) to determine their taxonomic identity. Multiple sequence alignment was performed using CLUSTAL W [32], and phylogenetic analysis was conducted using MEGA 11 version 11.0.12 to infer evolutionary relationships.

All molecular and proteomic identifications were performed at the PLAGENOR genomic platform.

Assessment of hydrolytic enzyme activities:

Amylase Activity

Amylase activity was screened using Starch Agar plates. Pure bacterial isolates were inoculated and streaked onto 1.5% starch agar plates, followed by incubation at 70 °C for 24 h. After incubation, the plates were treated with Gram’s Iodine crystals to form a deep blue starch-iodine complex. The presence of clear zones around the colonies indicated starch-degrading enzyme activity due to the hydrolysis of starch [33].

Laccase Production

Laccase activity was evaluated by testing the ability of bacterial strains to degrade Congo Red dye. The strains were first incubated in modified liquid medium (MLM) at 70 °C for 24 h. After incubation, cultures were standardized to an optical density (OD_600_) of 0.4, then 1 mL of each standardized culture was transferred into a similar medium supplemented with 100 mg/L Congo Red dye and incubated at 70 °C for six days. Laccase activity was determined by monitoring the decolorization of Congo Red over time [34].

DNase Activity

DNase activity was assessed using DNase Test Agar plates (Oxoid, Basingstoke, UK) supplemented with methyl green (0.05 g/L) as an indicator. Bacterial strains were inoculated onto the plates and incubated at 70 °C for 24 h. The formation of clear zones around bacterial growth indicated DNase activity [35].

Protease Activity

Protease production was evaluated using Skimmed Milk Agar (SMA) medium. each strain was first standardized to an optical density (OD_600_) of 0.5 then spot-inoculated onto SMA plates and incubated at 70 °C for 24 h. The hydrolysis of milk casein resulted in clear zones around the colonies, indicating protease activity [36].

To visualize the relationships between isolates based on their enzymatic profiles, a dendrogram was constructed using PAST software version 5.1, employing the UPGMA clustering method and the Jaccard coefficient.

Clearing Zone Intensity (*CI*) Quantification

For strains exhibiting positive enzymatic activity on agar plates, clearing zone intensity (*CI*) was quantified using the following formula [37]:

$$CI=\frac{\mathrm{Halo}\;\mathrm{zone}\;\mathrm{diameter}\;+\;\mathrm{Colony}\;\mathrm{diameter}}{\mathrm{Colony}\;\mathrm{diameter}}.$$

Each measurement was conducted in triplicate.

Statistical Analysis

Statistical analyses were carried out with GraphPad Prism version 8 using both one-way and two-way ANOVA, depending on the experimental design. Two-way ANOVA was used to determine significant differences between groups for physiological characterization (temperature and pH studies), while one-way ANOVA was employed to evaluate differences in enzymatic activities among strains. For amylase activity specifically, a *t*-test was conducted for paired comparisons. All statistical analyses used a significance threshold of *p* < 0.05. All experiments were conducted in triplicate to ensure reproducibility.

## 3. Results

Isolation and growth properties of thermophilic microorganisms:

The overall morphological characteristics of the eight isolated strains are summarized in Supplementary Table S1, while Supplementary Figure S2 shows the appearance of pure colonies grown on MSM agar plates.

**Figure 1:**
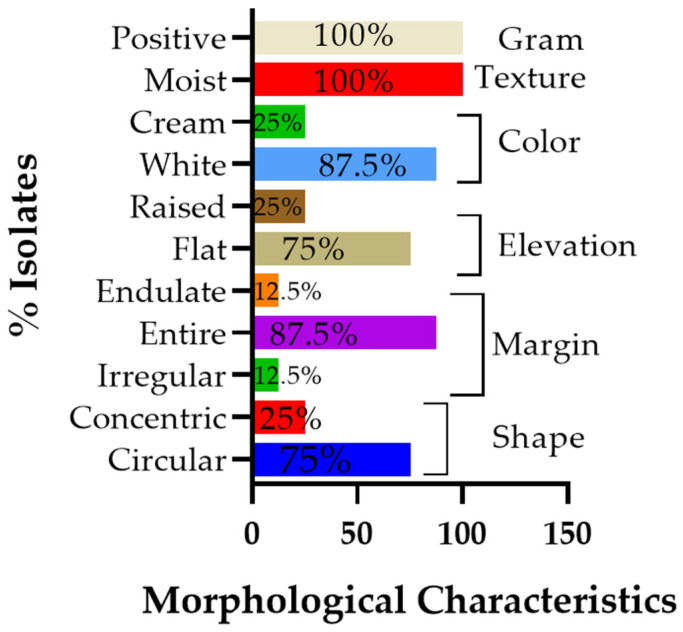
Horizontal bar chart showing the percentage distribution of principal morphological characteristics (gram reaction, texture, color, elevation, margin, and shape) among eight thermophilic bacterial isolates cultured on MSM agar plates.

Figure 1 below quantitatively illustrates the distribution of the key morphological criteria, displaying the percentage of isolates exhibiting each feature: gram reaction, texture, color, elevation, margin, and colony shape.

The colonies exhibited growth after 24 h of incubation at 70 °C. Most strains displayed whitish colony pigmentation, except for isolates AS01 and AS07, which had creamy color. Microscopic examination revealed that the cells were rod-shaped and Gram-positive. Additionally, the bacterial isolates retained viability after boiling treatment, as indicated by the turbidity of the growth media and quantified by changes in their optical density (OD) values measured within 48 h. Although the OD values varied (0.50, 0.30, 0.26, 0.30, 0.40, 0.32, 0.69, and 0.79) for strains AS01 to AS08, respectively, the persistence of growth in liquid medium confirmed their heat-resistant nature.

**Figure 2:**
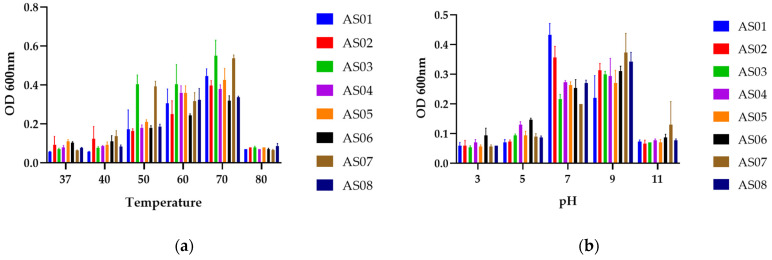
Bars represent the mean ± standard error of the mean (SEM) of strain growth at (**a**) different temperatures (37, 40, 50, 60, 70, and 80 °C at pH 8.4) and (**b**) pH ranges (3.0, 5.0, 7.0, 9.0, and 11.0 at 70 °C) after 48 h of incubation.

The isolates were examined for their tolerance to different temperatures and pH levels. All isolates exhibited an optimal growth temperature of 70 °C, Additionally, all strains demonstrated an optimal pH of 9 except AS01 and AS02 which had an optimal pH of 7. No growth was observed at 37 °C, further supporting their thermophilic nature. Similarly, growth was absent in highly acidic (pH 3.0–5.0) and extremely alkaline environments pH 11.0, as shown in Figure 2.

**Figure 3:**
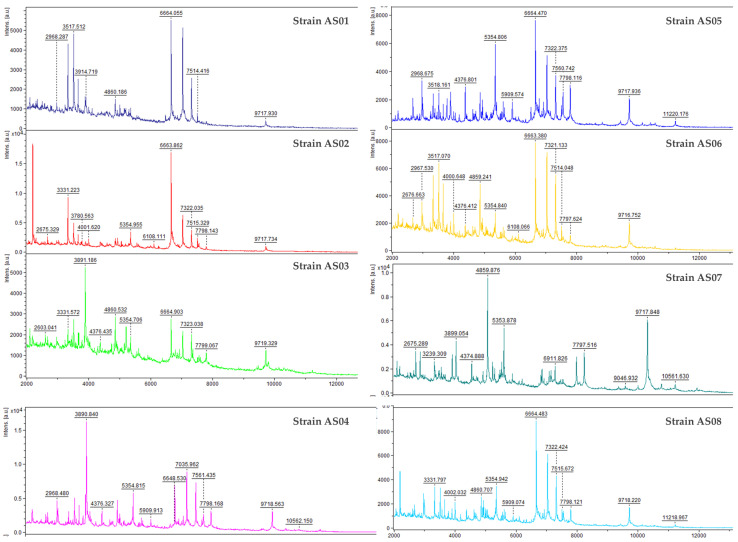
MALDI-TOF MS spectra of the eight isolated *Geobacillus* strains, illustrating characteristic mass profiles within the 2000 to 10,000 Da range. Specific peaks in the spectra highlight strain-dependent variations and conserved features among the strains.

MALDI-TOF MS identification of the isolates:

In this study, the eight isolates were analyzed using the MALDI Biotyper^®^ Sirius System at the PLAGENOR proteomics laboratory. The results revealed that six out of eight isolates were identified as *Geobacillus kaustophilus*, while two isolates were assigned to *Geobacillus jurassicus*, with identification scores presented in Table 1. However, an examination of the mass spectra revealed variations among all the isolates, those identified as *G*. *kaustophilus* and *Geobacillus jurassicus* (Figure 3). These results indicate that MALDI-TOF MS alone may not be sufficient for precise identification, as database limitations, particularly the underrepresentation of environmental *Geobacillus* strains in commercial reference libraries, can reduce accuracy. Therefore, complementary molecular techniques, such as genotyping and 16S rRNA sequencing, are needed to confirm the identity of the isolated strains.

BOX and RAPD-PCR fingerprinting:

In this study, two single oligonucleotides, BOX A1R and OPR13, were used to genotype the eight bacterial strains previously identified by MALDI-TOF MS. Both primers generated distinct banding profiles; however, PCR performed with OPR13 primer generated a significantly different pattern compared to BOX A1R.

**Figure 4:**
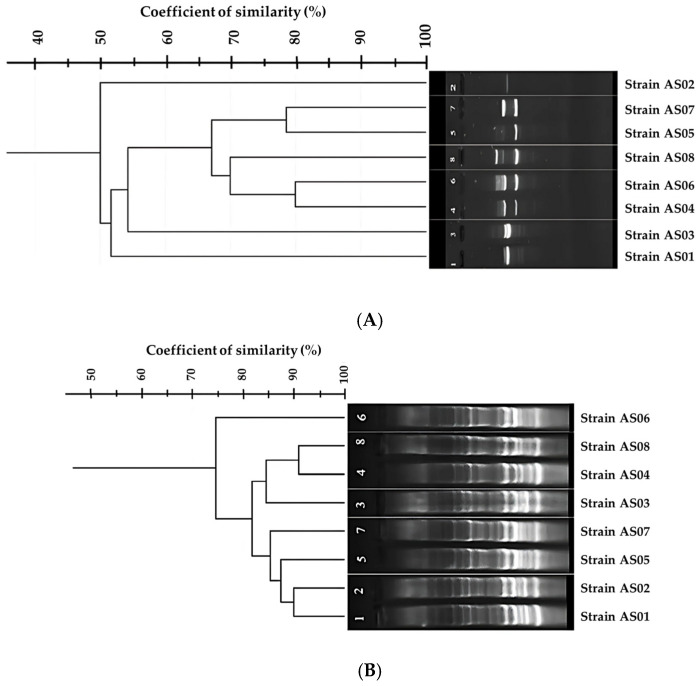
Genetic profiling of eight bacterial isolates using RAPD-PCR and BOX-PCR. Cluster analysis was performed based on Dice similarity coefficients using GelJ software (v2.0.0). (**A**) RAPD-PCR profile and corresponding dendrogram. (**B**) BOX-PCR profile and corresponding dendrogram.

The distinct banding patterns are shown in Figure 4, demonstrating significant genetic variation among the studied strains, which were identified as *Geobacillus kaustophilus* and *Geobacillus jurassicus* by MALDI-TOF MS, revealing intraspecific genomic diversity within these species, consistent with their proteomic profiles.

Figure 4 presents the BOX-PCR and RAPD-PCR profiles of the eight isolates, with cluster analysis based on Dice similarity coefficients performed using GelJ software (version 2.0.0).

The RAPD and BOX-PCR analyses revealed distinct clustering patterns among the eight strains. In the RAPD dendrogram, strains 2 and 8 formed a separate outgroup, while the main cluster comprised closely related pairs: (1,3), (4,6), and (5,7). Similarly, BOX-PCR analysis identified strains 3 and 6 as outgroup, with the remaining strains grouping into three clusters: (1,2), (5,7), and (4,8). These results demonstrate significant intraspecific genetic diversity, with clear phylogenetic separation between outgroups and main clusters. The observed patterns reflect evolutionary divergence within the strains studied.

16S rRNA sequencing:

To refine the preliminary identifications obtained via MALDI-TOF MS and establish phylogenetic relationships (Table 2), 16S rRNA gene sequencing was performed for all eight isolates using universal primers, as described in the materials and methods section.

Pairwise sequence comparisons of the 16S rRNA genes were conducted using the Maximum Composite Likelihood method implemented in MEGA version 11. The resulting genetic distance matrix is presented in Supplementary Table S2.

**Figure 5:**
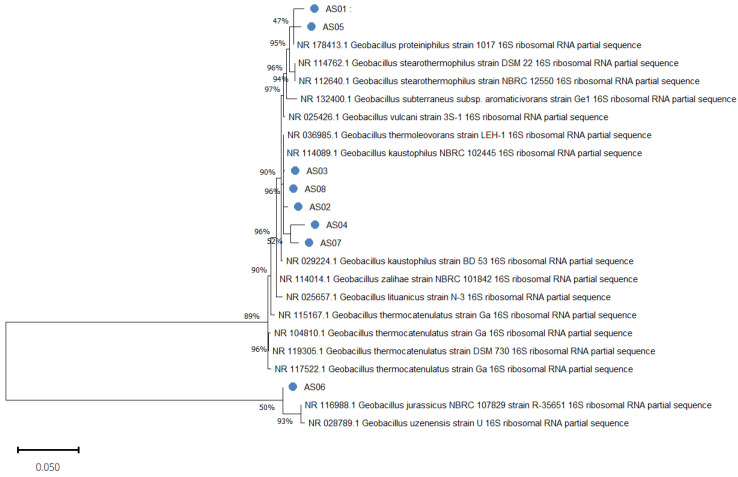
Phylogenetic analysis of the eight bacterial isolates along with their closest related type strains, based on 16S rRNA gene sequences. The phylogenetic tree was constructed using the maximum likelihood method with the Jukes–Cantor model in MEGA 11. Bootstrap values are shown at the branch points. The scale bar represents 0.05 nucleotide substitutions per site.

The phylogenetic tree (Figure 5) was constructed based on the 16S rRNA gene sequences of the eight isolates alongside closely related reference strains from the NCBI GenBank database, using the maximum likelihood method. Bootstrap support values greater than 45% are shown at the nodes, indicating the reliability of the branching patterns.

The analysis revealed that all isolates clustered within the genus *Geobacillus*, confirming their affiliation as previously indicated by MALDI-TOF MS results, as shown in Table 1. The isolates segregated into distinct clades, reflecting intra-genus diversity. AS01 and AS05 formed a separate cluster with relatively low bootstrap support (47%), closely related to *Geobacillus proteiniphilus*. A robust clade (bootstrap support ≥ 90%) included AS03, AS08, and AS02, which clustered with *Geobacillus kaustophilus* and *Geobacillus thermoleovorans* strains. AS04 and AS07 grouped together within the same lineage but formed a separate branch, while AS06 formed an independent branch, showing 100% identity to *Geobacillus kaustophilus* yet clustering within the *G. kaustophilus/thermocatenulatus* lineage, with moderate bootstrap support (50%).

Pairwise analysis of the 16S rRNA gene sequences among the eight *Geobacillus* isolates revealed a very high level of sequence similarity, with genetic distances ranging from 0.0008 (0.08%) between AS06 and AS03 to 0.0134 (1.34%) between AS07 and AS04, as calculated using the Maximum Composite Likelihood method in MEGA version 11. The complete pairwise distance matrix is provided in Supplementary Table S2. These small genetic distances strongly confirm that the isolates are closely related at the species level and likely belong to a group of very closely related species.

This genetic closeness is consistent with the phylogenetic analysis based on 16S rRNA gene sequences (Figure 5), which clustered the isolates into distinct but related clades within the *Geobacillus* genus.

Assessment of enzyme-producing potential in bacterial isolates:

The capacity of each isolated strain to produce extracellular thermostable hydrolytic enzymes, including protease, amylase, laccase, and DNase, was assessed at 70 °C. All eight bacterial isolates exhibited at least one enzymatic activity when tested. Amylase, DNase and protease activities were detected qualitatively on solid media, whereas laccase activity was observed via decolorization in liquid medium.

Screening revealed varying capabilities among the isolates; specifically, amylolytic activity was detected in two out of eight strains (25%), while proteolytic activity was observed in five out of eight strains (62.5%). In contrast, both DNase and laccase activities were detected in all isolates (100%).

**Figure 6:**
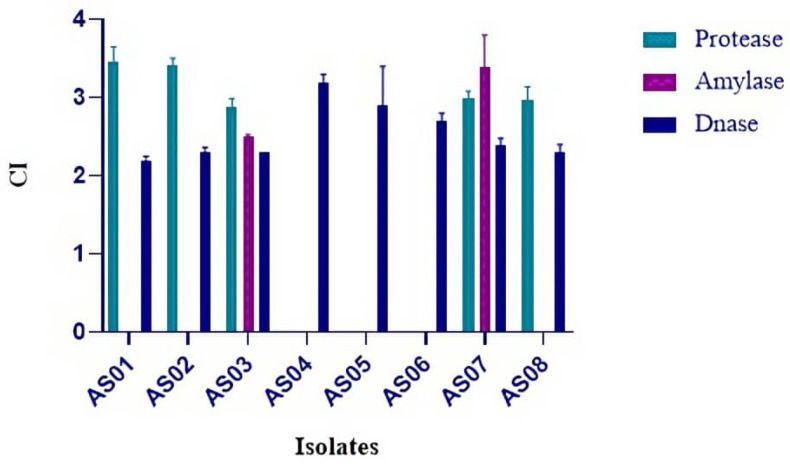
The clearing zone intensities (*CI*) of protease, amylase, and DNase activities were measured for eight bacterial isolates. The data are presented as mean ± standard error (SE) based on triplicate experiments. The error bars represent the standard error of the mean.

Quantitative assessment based on clearing zone intensity (*CI*) further demonstrated variability in the production levels of protease, amylase, and DNase. Protease activity (*CI*) values ranged from 2.88 ± 0.11 to 3.45 ± 0.2, indicating relatively minor variations among the producing strains. Amylase activity was detected exclusively in strains AS03 and AS07, with the highest recorded CI reaching 3.4 ± 0.4. DNase activity (*CI*) ranged from 2.2 ± 0.05 to 3.2 ± 0.1. Statistically significant differences were observed in DNase activity among the strains (one-way ANOVA, *p* = 0.04), although the overall variation was less pronounced compared to protease activity (Figure 6).

**Figure 7:**
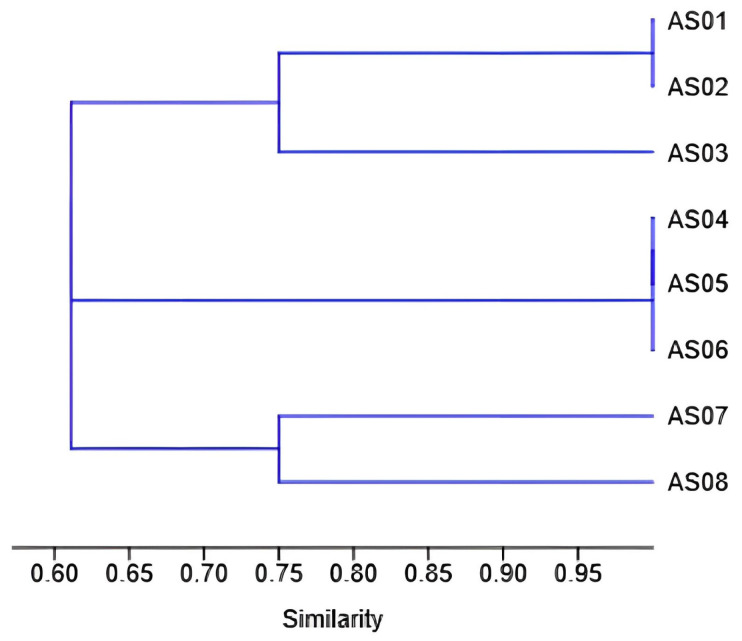
Dendrogram-based classification of the eight isolates based on their enzymatic activities using UPGMA clustering and Jaccard coefficient.

Clustering analysis of the isolated strains based on their phenotypic characteristics, particularly their ability to produce thermostable enzymes, was performed. The clustering analysis grouped the isolates into three distinct clusters based on their enzymatic activity using the UPGMA method (Figure 7). Cluster A included AS01, AS02, and AS03, with AS03 showing a slight distinction from the first two. Cluster B consisted of AS04, AS05, and AS06, sharing a high degree of similarity. The final cluster contained AS07 and AS08, each positioned on a separate branch, reflecting their relatedness.

## 4. Discussion

Microorganisms with an optimal growth temperature between 60 °C and 80 °C are classified as thermophiles [38]. Geothermal zones are considered the primary source of these microorganisms [39]. their diversity has been extensively studied in hot springs, as these environments provide optimal conditions for their growth and extreme adaptation [23,40]. Although thermophiles are predominantly found in high-temperature environments, they can also be present in sandy soils as reported by Al-Mulla et al. [41]. Algeria, as one of the largest countries with numerous thermal springs, has been the subject of several studies that successfully isolated and identified thermophilic bacterial species, including archaea [36,42,43]. However, no studies have yet been conducted to identify thermophiles present in desert sand from Algerian arid regions. Despite the low habitability of these environments due to unfavorable conditions, certain microorganisms have adapted to these extreme habitats, finding them optimal for growth and reproduction.

In this study, extreme thermophilic bacteria were isolated from the sandy soil of the Algerian Sahara. A total of eight aerobic strains were obtained, all exhibiting a rod-shaped morphology, Gram-positive staining, and endospore-forming capability. These morphological traits represent key adaptive mechanisms that enable thermophiles to survive in extreme environments, contributing to the metabolic and ecological versatility of prokaryotic taxa [41] with endospore formation conferring resistance to high temperatures [44]. These phenotypic characteristics were observed in *Geobacillus yumthangensis* sp. nov. strain *AYN2*, a thermophilic bacterium isolated from a hot spring in northeastern India [45], as well as in thermophiles recovered from diverse ecological niches, including boiled cow milk, composted manure, and tomato rhizospheric soil [46]. Similarly, Rong Tang et al. reported the isolation of two novel thermophilic strains from mangrove sediment, exhibiting identical morphological traits [47]. These phenotypic attributes play a critical role in the survival and adaptability of thermophilic species in extreme habitats.

The growth characteristics of the isolates across temperatures ranging from 37 °C to 80 °C and pH values from 3 to 11 are presented in Figure 2. All results were statistically significant, with a *p*-value below 0.05 for both analyses (0.002 for temperature and 0.0001 for pH), confirming that both temperature and pH significantly influence bacterial growth across the isolates [48]. These bacteria exhibited growth within a temperature range above 40 °C, confirming their thermophilic nature [49], with an optimal growth temperature of 70 °C, thereby confirming their classification as obligate thermophiles as discussed by Zeigler [50]. Moreover, the production of metabolites occurs under specific temperature and pH conditions optimal for them; otherwise, metabolite inhibition occurs, leading to growth inhibition [23]. These findings are closely matching with those reported by Sharma et al. [51], which demonstrated an optimal temperature range of 65–70 °C and a pH range of 6–8 of their isolates.

Several techniques have been established in the literature and continue to be used for microbial identification, primarily based on PCR reactions. Among these are genotyping techniques such as RAPD, ARDRA, and RISA, which must be systematically followed by 16S rRNA gene sequencing to ensure the validity and accuracy of the results [52]. MALDI-TOF MS has been extensively used for bacterial identification and is now a key tool in scientific research, particularly for the identification of extremophilic species [53,54]. This method compares the protein profiles of isolates with those in reference databases to determine the species under investigation [55]. Although MALDI-TOF MS can accurately differentiate many bacterial species, its efficiency decreases when closely related species exhibit highly similar protein profiles [56].

The BOX-PCR results supported the MALDI-TOF MS findings, as the generated profiles show low discriminatory similarity, indicating that BOX primers were unable to effectively discriminate between the strains. Although BOX-PCR has been reported to provide superior discrimination for thermophilic lactic acid bacteria associated with dairy products [57] and for species identification within the genus *Bacillus* [58], our results showed discordance with these findings, as the BOX A1R primer failed to achieve a high level of strain differentiation. Nevertheless, cluster analysis based on banding profile similarity grouped the isolates into distinct genotypic clusters despite the limited discriminatory power of these methods. To further assess genetic variation among the isolates, RAPD-PCR was employed, a technique widely recognized for its ability to differentiate closely related species [59]. Following RAPD-PCR analysis, a clear distinction among the isolated strains was observed, with cluster analysis revealing discrete genotypic groupings, evidenced by distinct banding profiles in the electrophoretic patterns, indicating its effectiveness in bacterial genotyping. RAPD-PCR is considered one of the reliable methods for bacterial differentiation, as supported by previous studies [29,60] where six *Bacillus* strains isolated from Cheonggukjang “fermented soybean paste.” were identified at the species level, an identification that was challenging using 16S rRNA and recA gene sequencing. Additionally, RAPD-PCR has been successfully applied for genotyping thermophilic bacterial pathogens [61], further demonstrating its utility in differentiating closely related bacterial strains. Genomic variation among strains of the same species, known as intraspecific genetic diversity, was detected using two genotyping techniques, BOX-PCR and RAPD-PCR. Both methods reveal this diversity by generating distinct genomic fingerprints that reflect differences in the distribution of primer-binding sites between them.

In BOX-PCR, the single primer is designed to anneal to conserve sequences within BOX repetitive elements dispersed throughout the bacterial genome. While BOX-PCR profiles of the strains from the same species display a set of shared core bands representing conserved genomic regions, they also show differences in the number and position of additional bands, such differences are typically caused by genomic rearrangements, insertions, deletions, or horizontal gene transfer events [62]. In RAPD-PCR, short random primers anneal to multiple complementary sequences across the genome under low-stringency conditions [63], with amplification occurring only when two primer-binding sites are located on opposite strands and within an amplifiable distance. Polymorphisms such as single nucleotide substitutions, insertions, deletions, and structural rearrangements can create or eliminate primer-binding sites, thereby altering the number and size of amplified fragments [64]. These genomic variations generate strain-specific banding patterns within a single species. In this study, RAPD-PCR revealed a higher degree of profile heterogeneity compared to BOX-PCR, characterized by unique amplicons and distinct presence/absence patterns. Cluster analyses based on Dice similarity coefficients demonstrated the existence of measurable intraspecific genetic separation among the isolates, confirming the utility of RAPD-PCR for detecting fine-scale genomic diversity.

Molecular identification of the isolates based on the 16S rRNA gene confirmed their affiliation with the *Geobacillus* genus, as previously determined by MALDI-TOF MS. To achieve species-level classification, the maximum likelihood (ML) method was employed due to its efficiency in phylogenetic tree construction [65]. Phylogenetic analysis revealed that the isolates named AS01 and AS05 exhibited similarity to *Geobacillus proteiniphilus*, sharing several morphological traits with those reported by Semenova et al. [66], with variations observed between the strains, likely reflect adaptive responses to their specific environmental conditions. Microorganisms, including extremophiles, display environment-dependent physiological and metabolic changes, which the same strain can behave differently in distinct extreme environments [67]. Also, climatic fluctuations have been identified as a key driver species divergence formation [68], which may account for this variation. Notably, the isolates in this study were obtained from Algerian sand, whereas the other *G. proteiniphilus* was isolated in China from heavy oil reservoir, our isolates exhibited an optimal growth temperature of 70 °C, whereas the reference *G. proteiniphilus* demonstrated optimal growth between 60 °C and 65 °C, as documented in the BACDIVE database (https://bacdive.dsmz.de/strain/166233, accessed on 1 March 2025), potentially contributing to strains variations.

The remaining strains were taxonomically assigned to other *Geobacillus* species (*G. kaustophilus*, *G. thermoleovorans*, and *G. thermocatenulatus*), revealing significant intraspecific heterogeneity. This observed diversity, even among conspecific isolates, likely reflects evolutionary adaptations of thermophilic strains, as shown in a study of *Geobacillus stearothermophilus*, a species traditionally characterized by an inability to utilize lactose. However, recent findings have shown that dairy-associated strains of *G. stearothermophilus* have acquired the ability to metabolize lactose, showing the evolutionary adaptability of the species [69], which reinforces the idea of the evolution according to the environmental conditions from which the isolates were recovered [70]. These observations reinforce the hypothesis that genetic discrepancies among closely related strains can lead to functional differentiation over time which supports the idea that genetic divergence through evolutionary processes influences phylogenetic clustering [71].

The screening of thermozymes production revealed that all the isolates are producers of DNase and laccase at varying levels. DNase production was confirmed to be statistically significant, with a *p*-value < 0.05 based on the triplicate measurements of clearance intensity, while laccase production was validated by the rate of dye decolorization, which was visually noticeable. These two enzymes have been shown to be produced by thermophiles, according to studies reported by [12,72]. Amylase production was observed in only two strains, while protease production was detected in five strains. The lack of statistical significance in protease between the studied strains and amylase between the same strains was due to the close enzymatic intensities among the producing isolates, indicating no substantial difference in their enzyme production levels.

A previous study [73] reported that a high percentage of the isolates exhibited high amylase and protease enzymatic activity. However, in our study, a difference was observed regarding amylase production, as the previous screening was conducted at 45 °C, while ours was performed at 70 °C. This temperature variation may influence enzyme structure, stability, and activity at high temperatures. A similar result was observed in another study [42], which found that most of their strains produced amylase and protease when screened at 55 °C. Furthermore, the results obtained by Thebti et al. [74] show a high degree of similarity with our findings, as their enzymatic screening at 70 °C revealed that only a few strains exhibited Amylase and protease activity at this temperature, supporting the validity of our results.

The potential of these isolates to produce thermostable enzymes at high temperatures, while others did not, can be attributed to differences in enzyme stability mechanisms. Several studies have demonstrated that the stability temperature of thermozymes is higher than their production temperature. For instance, in a study conducted by Nand et al. [75], an endoxylanase production was screened at 65 °C, whereas the enzyme exhibited stability up to 100 °C, with optimal activity at 70 °C. Similarly, another study reported that an alkaline protease produced by *Thermus thermophilus* showed high stability at 80 °C, despite being produced at 70 °C [76]. This thermostability is influenced by the number of hydrogen bonds, disulfide bridges, salt bridges, and the presence of hydrophobic amino acids [77,78], demonstrating remarkable thermal stability that surpasses mesophilic enzymes [79]. Another feature of these thermozymes is their resistance to chemical denaturants, extreme pH, and organic solvents, making them more robust in diverse industrial environments compared to mesozymes [80], also operating at higher temperatures reduces microbial contamination risk and lowers viscosity of reaction mixtures, a key features of thermozymes [81]. All these characteristics make them promising candidates, particularly in biotechnology sectors requiring robust enzymatic tools for industrial applications [4] like in industrial biomass conversion [82] and in biofuel production through lignocellulosic saccharification [83]. Multiple studies have identified *Geobacillus* species with industrially relevant enzymatic activities, further emphasizing their biotechnological potential due to their ability to produce thermostable enzymes at elevated temperatures [84,85,86].

The study of extremophilic species in an unexplored environment, as in our case, represents a valuable strategy for discovering interested extremophilic bacterial species. This exploration is inherently linked to the presence of thermozymes with unique characteristics compared to their parental strains, exhibiting promising potential for biotechnological applications that demand high-temperature enzymatic activity and stability.

These findings highlight the need for further in-depth studies to confirm strain identity, particularly through whole-genome sequencing of these isolates, to explore their thermostable enzymatic repertoire. Due to the remarkable properties of these enzymes, such as low risk of contamination, high catalytic efficiency at elevated temperatures, and increased process speed, they hold great potential for various biotechnological applications. Harnessing these resources could open new opportunities in industries such as food processing, bioremediation, and the production of thermostable enzymes for biotechnological applications.

## Figures and Tables

**Table 1:** MALDI-TOF MS-based identification and reliability score of the bacterial isolates.

Isolates Code	Detected Species	MALDI-TOF Score
AS01	*Geobacillus kaustophilus*	1.91
AS02	*Geobacillus kaustophilus*	2.25
AS03	*Geobacillus jurassicus*	1.86
AS04	*Geobacillus kaustophilus*	1.98
AS05	*Geobacillus kaustophilus*	2.02
AS06	*Geobacillus kaustophilus*	2.14
AS07	*Geobacillus jurassicus*	1.98
AS08	*Geobacillus kaustophilus*	2.13

**Table 2:** Phylogenetic affiliations of the isolated strains based on 16S rRNA gene sequence analysis, including GenBank accession numbers of reference strains and sequence similarity percentages.

Strain	Closest Species	% Similarity	Accession Number
AS01	*Geobacillus proteiniphilus* strain 1017	99.46%	NR_178413.1
AS02	*Geobacillus kaustophilus* NBRC 102445	99.03%	NR_114089.1
AS03	*Geobacillus kaustophilus* NBRC 102445	99.87%	NR_114089.1
AS04	*Geobacillus thermoleovorans* strain LEH-1	97.98%	NR_036985.1
AS05	*Geobacillus proteiniphilus* strain 1017	98.26%	NR_178413.1
AS06	*Geobacillus kaustophilus* NBRC 102445	100%	NR_114089.1
AS07	*Geobacillus kaustophilus* NBRC 102445	98.91%	NR_114089.1
AS08	*Geobacillus thermoleovorans* strain LEH-1	99.86%	NR_036985.1

## Data Availability

Data are contained within the article and Supplementary Materials.

## Supplementary Materials

The following supporting information can be downloaded at https://www.mdpi.com/article/10.3390/life15081327/s1: Supplementary Figure S1: Study area map showing the sandy soil sampling site (indicated by the yellow dot) in the desert ecosystem of Aïn Sefra, Naâma Province, Algeria (32°44′18.0″ N 0°34′59.0″ W); Supplementary Table S1: Morphological characteristics of the isolated strains; Supplementary Figure S2: Macro observation of the morphology of the eight bacterial isolates recovered from sand dunes, cultured on MSM at 70 °C; Supplementary Table S2: Pairwise differences between 16S rRNA gene sequences of the studied strains calculated using the maximum composite likelihood method.
