# Supplementary material for: Integrated Proteomic and Molecular Identification of Thermophilic Geobacillus Strains from Algerian Desert Sands and Their Enzymatic Potential
Source: Life (Basel). 2025 Aug 21;15(8):1327. doi: 10.3390/life15081327 (PMC12387256; doi:10.3390/life15081327)
Supplement: Supplementary file 1 [file life-15-01327-s001.zip › life-3799089-supplementary.pdf]

life

MDPI

Article

Integrated Proteomic and Molecular Identification of Thermophilic *Geobacillus* Strains from Algerian Desert Sands and Their Enzymatic Potential

Amaria Ilhem Hammadi [1], Mohamed Merzoug [1*], Marwa Aireche [1], Zohra Yasmine Zater [2], Keltoum Bendida [1], Chaimaa Naila Brakna [1], Slimane Choubane [1], Svetoslav Dimitrov Todorov [3,4,*] and Djamal Saidi [1]

1. Higher School of Biological Sciences of Oran, BP 1042 Saim Mohamed, Cité Emir Abdelkader (EX-INESSMO) 31000 Oran, Algeria. hammadiamaria267@gmail.com (A.I.H.); merzoug.mohamed1@yahoo.fr (M.M.); marwa.ar231@gmail.com (M.A.); keltoumbendida2001@gmail.com (K.B.); chaimaa1012@gmail.com (C.N.B.); choubane.slimane@gmail.com (S.C.); djamsaidi@gmail.com (D.S.)
2. Laboratory of Biology of Microorganisms and Biotechnology, University of Oran1 Ahmed Ben Bella, Oran, Algeria 31005. yassminezater93@gmail.com (Z.Y.Z.)
3. ProBacLab, Laboratório de Microbiologia de Alimentos, Departamento de Alimentos e Nutrição Experimental, Food Research Center, Faculdade de Ciências. Farmacêuticas, Universidade de São Paulo, São Paulo 05508-000, SP, Brazil. slavi310570@abv.bg; todorov@usp.br (S.D.T.)
4. Department of General Hygiene, I.M. Sechenov First Moscow State Medical University, Trubetskaya St., Bldg. 8/2, Moscow 119435, Russia.

* Correspondence: merzoug.mohamed1@yahoo.fr; slavi310570@abv.bg

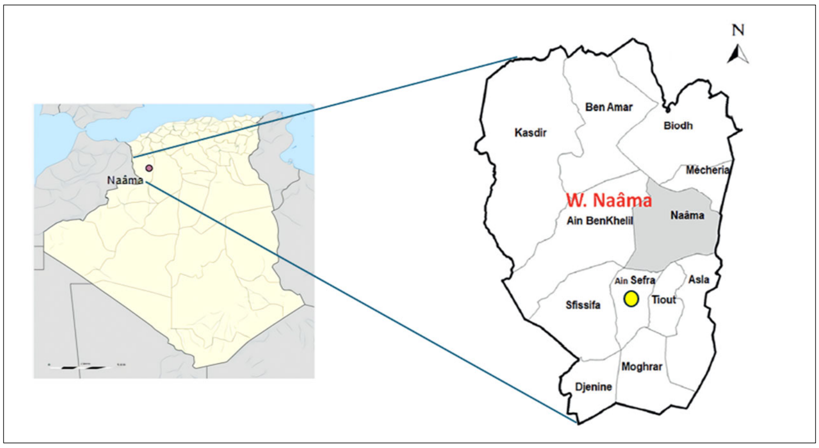

Supplementary Figure S1. Study area map showing the sandy soil sampling site (indicated by the yellow dot) in the desert ecosystem of Ain Safra, Naâma Province, Algeria (32°44′18.0″N 0°34′59.0″W).

Supplementary Table S1. Morphological characteristics of the isolated strains.

Isolates.	Shape	Margin	Elevation	Color	Texture	Gram
**AS01**	Circular	Entire	Flat	Cream	Moist	Positive
**AS02**	Circular	Entire	Raised	White	Moist	Positive
**AS03**	Concentric	Entire	Flat	White	Moist	Positive
**AS04**	Circular	Entire	Flat	White	Moist	Positive
**AS05**	Irregular	Undulate	Flat	White	Moist	Positive
**AS06**	Circular	Entire	Flat	White	Moist	Positive
**AS07**	Concentric	Entire	Flat	Cream	Moist	Positive
**AS08**	Circular	Entire	Raised	White	Moist	Positive

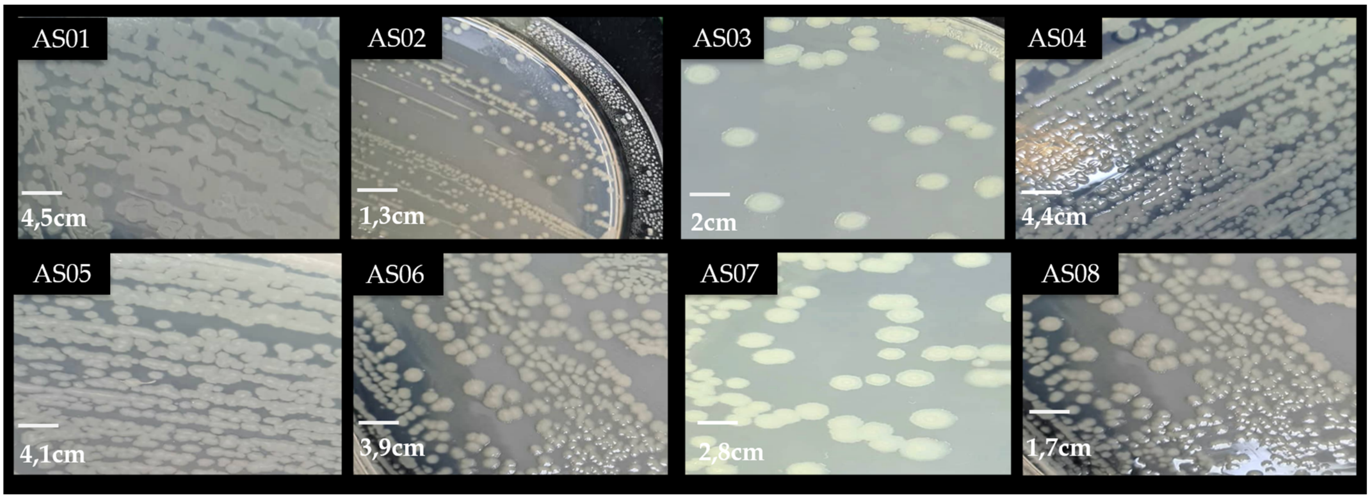

Supplementary Figure S2. Macro observation of the morphology of the eight bacterial isolates recovered from sand dunes, cultured on MSM medium at 70°C

Supplementary Table S2. Pairwise differences between 16S rRNA gene sequences of the studied strains calculated using the maximum composite likelihood method.

	AS01	AS02	AS03	AS04	AS05	AS06	AS07	AS08
**AS01**	-	0.0026	0.0025	0.0118	0.0055	0.0017	0.0025	0.0018
**AS02**	0.0026	-	0.0034	0.0122	0.0066	0.0026	0.006	0.0030
**AS03**	0.0025	0.0034	-	0.0108	0.0037	0.0008	0.0055	0.0009
**AS04**	0.0118	0.0122	0.0108	-	0.0110	0.0099	0.0134	0.0106
**AS05**	0.0055	0.0066	0.0037	0.0110	-	0.0054	0.0082	0.0069
**AS06**	0.0017	0.0026	0.0008	0.0099	0.0054	-	0.009	0.0000
**AS07**	0.0025	0.006	0.0055	0.0134	0.0082	0.0049	-	0.0035
**AS08**	0.0018	0.003	0.0009	0.0106	0.0069	0.0000	0.0035	-
